# Association between Lead and Cadmium and Reproductive Hormones in Peripubertal U.S. Girls

**DOI:** 10.1289/ehp.1001943

**Published:** 2010-07-30

**Authors:** Audra L. Gollenberg, Mary L. Hediger, Peter A. Lee, John H. Himes, Germaine M. Buck Louis

**Affiliations:** 1 Division of Epidemiology, Statistics and Prevention Research, *Eunice Kennedy Shriver* National Institute of Child Health and Human Development, Rockville, Maryland, USA;; 2 Department of Pediatrics, Penn State College of Medicine, Hershey, Pennsylvania, USA;; 3 Division of Epidemiology and Community Health, University of Minnesota School of Public Health, Minneapolis, Minnesota, USA

**Keywords:** heavy metals, inhibin B, luteinizing hormone, NHANES, puberty

## Abstract

**Background:**

Lead (Pb) and cadmium (Cd) are known reproductive toxicants thought to disrupt hormone production throughout sensitive developmental windows, although this has not been previously examined in nationally representative peripubertal children.

**Objectives:**

We examined the association between blood Pb and urinary Cd concentrations and the reproductive hormones inhibin B and luteinizing hormone (LH) in girls 6–11 years of age who participated in the cross-sectional Third National Health and Nutrition Examination Survey (NHANES III) (1988–1994).

**Methods:**

Pb (micrograms per deciliter) was measured in whole blood, and Cd was measured in urine (nanograms per milliliter). Inhibin B (picograms per milliliter) and LH (milli–International units per milliliter) were measured in residual sera for 705 girls. Survey logistic regression was used to estimate associations with pubertal onset based on inhibin B concentration > 35 pg/mL or LH concentration > 0.4 mIU/mL, and multinomial logistic regression was used to estimate the association between Pb and increasing categories of hormone concentrations.

**Results:**

High Pb (≥ 5 μg/dL) was inversely associated with inhibin B > 35 pg/mL [odds ratio (OR) = 0.26; 95% confidence interval (CI), 0.11–0.60; compared with Pb < 1 μg/dL]. At 10 and 11 years of age, girls with low Pb (< 1 μg/dL) had significantly higher inhibin B than did girls with moderate (1–4.99 μg/dL) or high Pb (≥ 5 μg/dL). In the subsample of 260 girls with levels of inhibin B above the level of detection and using survey regression modeling, inhibin B levels were lower among girls with both high Pb and high Cd (β = −0.52; 95% CI, −0.09 to −1.04) than among girls with high Pb alone (β = −0.35; 95% CI, −0.13 to −0.57), relative to girls with low Pb and low Cd.

**Conclusions:**

Higher Pb was inversely associated with inhibin B, a marker of follicular development, and estimated effects suggestive of pubertal delays appeared to be stronger in the context of higher Cd concentrations. These data underscore the importance of Pb and Cd as reproductive toxicants for young girls.

The onset of puberty is a milestone marked by the appearance and development of secondary sexual characteristics and changes in behavior, growth, and reproductive capabilities. Changes in the timing of pubertal onset and/or progression have been associated with nutrition, obesity, and environmental contaminants, such as heavy metals and endocrine disruptors, supporting an environmental etiology to some degree (Buck Louis et al. 2008; Euling et al. 2008; Parent et al. 2005). Changes in the timing of onset and/or progression of puberty on a national scale have considerable public health and social implications for both boys and girls (Herman-Giddens 2006). Girls who mature relatively earlier are at increased risk of later obesity, diabetes, and reproductive-site cancers (Golub et al. 2008), and early-maturing girls and boys are reported to engage in more risky behaviors, such as cigarette smoking, substance use, and sexual activity (Dunbar et al. 2008; Hayatbakhsh et al. 2009). Relatively late-maturing girls, on the other hand, are at risk for diminished bone strength and fragility fractures later in life (Chevalley et al. 2009), whereas late-maturing boys may be at risk for victimization and depressive symptoms (Michaud et al. 2006). Assessment of environmental influences on the timing of puberty is thus important for health across the human life span, whether such influences are associated with advancements or delays in the onset of puberty.

However, few population-based data are available to characterize either environmental exposures or pubertal onset, contributing to our inability to identify environmental threats to human development across such sensitive windows. In girls, studies have relied primarily on physical signs, such as sexual maturation staging (Tanner 1962), or age at menarche as indicators of pubertal status and timing, although such signs are better indicators of later pubertal development than of onset (Denham et al. 2005; Selevan et al. 2003; Wu et al. 2003). At the population level, and despite claims of a declining secular pattern for pubertal onset, few studies have incorporated hormonal markers, although these are the clinical standard. Such studies are few perhaps because of the low expected concentrations in young children, the diurnal patterns of hormonal secretion, and the insensitivity of most hormones as markers. To address this critical data gap, we implemented a hormonal follow-on study for a representative sample of girls 6–11 years of age who participated in the Third National Health and Nutrition Examination Survey (NHANES III; 1988–1994) and for whom blood lead (Pb) and urinary cadmium (Cd) concentrations were determined (Brody et al. 1994; Paschal et al. 2000; Pirkle et al. 1994). We measured concentrations of two reproductive hormones—inhibin B and luteinizing hormone (LH)—believed to be relevant for younger girls (6–11 years of age) near the onset of puberty and that serve as markers of hypothalamic–pituitary–gonadal functioning. We are unaware of any other research aimed at assessing the effect of environmental chemicals or metals in relation to pubertal hormonal markers to delineate possible pathways of action.

## Methods

### Study participants and available measures

We used data from girls 6–11 years of age who participated in NHANES III, which collected data from 1988 through 1994. NHANES III is a cross-sectional, nationally representative survey with a complex multistage clustered probability sampling design. Blacks and Mexican Americans were oversampled to provide more precise estimates for these groups, and children were sampled into the age categories < 6, 6–11, and 12–19 years. Study design details and data collection methods have been previously published (National Center for Health Statistics 1994).

We included 705 girls 6–11 years of age at the time of screening who participated in the household interview (44% of the 1,589 girls in the 6- to 11-year age range) and who *a*) had a physical examination, including a standardized anthropometric assessment (National Center for Health Statistics 1994), and *b*) had residual stored biospecimens for laboratory analysis. Blood was drawn from the children at the time of clinical examination according to NHANES III protocol and categorized as to timing of blood draw (morning, 0800–1200 hours; afternoon, 1200–1600 hours; evening, 1600–1900 hours).

At 8–11 years of age, assessments were also made of pubertal status (Tanner 1962). Tanner staging for pubic hair and breast development classifies girls into progressive stages, ranging from stage 1 (no pubertal development) to stage 5 (fully mature). Using the NHANES III reliability data in a separate analysis, we determined that the reliability of Tanner staging was high [i.e., intraclass correlation coefficient, 0.86; 95% confidence interval (CI), 0.77–0.92; *n* = 44] for girls 8–11 years of age. Nevertheless, Tanner staging was not a focus of the present study because this pubertal assessment was limited to children 8–11 years of age, whereas hormone measurements were available for all children 6–11 years of age with available stored sera.

Several of the other relevant covariates were obtained from the NHANES III database. Sociodemographic variables of interest included race/ethnicity (non-Hispanic white, non-Hispanic black, Mexican American, other), census region (Northeast, Midwest, South, West), metropolitan/nonmetropolitan residence, and the poverty–income ratio (PIR). The PIR was computed as the total household income divided by the poverty threshold for the year of the interview (U.S. Department of Agriculture, Food and Nutrition Service Financial Management and Program Information Division 1987). The poverty threshold is determined annually by the U.S. Bureau of Census taking into account geographic location, rate of inflation, and family size. Based on the distribution of PIR (range, 0–6.9; median, 1.3), we categorized it as < 1, 1–2, and > 2, so that a higher PIR indicates better household financial status.

Other biological covariates examined as potential confounders included measures of iron deficiency, exposure to environmental tobacco smoke (Kolasa et al. 1998), and anthropometric measurements, which could affect both heavy metal body burden and pubertal development. Iron deficiency at examination was defined as two of three measures below age-specific cutoff values for transferrin saturation, serum ferritin, and erythrocyte protoporphyrin according to the guidelines provided by the Centers for Disease Control and Prevention (CDC) (Looker et al. 1997). Because only two (< 0.1%) girls reported smoking, we did not assess that exposure further, but we did assess the effect of environmental tobacco smoking exposure, which we defined as living with a cigarette smoker (yes/no).

### Laboratory analysis

Pb was measured in whole blood (micrograms per deciliter), and Cd (nanograms per milliliter) was measured in urine using established standards of practice (Brody et al. 1994; Gunter et al. 1996). The spectrometric laboratory methods used by NHANES III to measure Pb and Cd are universally accepted methods that yield adequate sensitivity at lower detection levels and use small volumes of biological sample (Lemos and de Carvalho 2009). Nonfasting serum samples that were stored at −70°C were obtained from the National Center for Health Statistics/CDC, and hormonal assays (inhibin B and LH) were performed by Rules-Based Medicine, Inc. (Austin, TX). Inhibin B was measured using the DSL-10-84100 ACTIVE Inhibin B enzyme-linked immunosorbent assay (ELISA; Gen I assay, Diagnostic Systems Laboratories, Inc., Webster, TX), an enzymatically amplified two-site two-step sandwich-type immunoassay with excellent internal validity [see Supplemental Material, Technical Note 1 (doi:10.1289/ehp.1001943)]; concentration was calculated from the standard curve. The functional sensitivity was *a priori* set at 7 pg/mL [i.e., limit of detection (LOD)] per manufacturer instructions. Because values below the LOD were not accessible, these were substituted uniformly with 
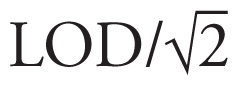
. LH was measured using the LH ELISA kit (Bio-Quant BQ049F; Bio-Quant, Inc., San Diego, CA), a solid-phase direct sandwich method [see Supplemental Material, Technical Note 1 (doi:10.1289/ehp.1001943)]. The functional sensitivity (LOD) was set at 0.05 mIU/mL, and values below these levels were uniformly substituted with 0.035 mIU/mL, equivalent to 
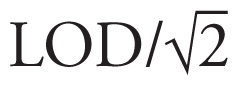
.

We chose *a priori* the hormone cutoffs that were most consistent with a pubertal individual given the limited available literature (inhibin B > 35 pg/mL and LH > 0.4 mIU/mL) (Crofton et al. 2002; Houk et al. 2009; Sehested et al. 2000; Teilmann et al. 2007). We acknowledge that there are no universal criteria for setting cutoffs to discriminate between pubertal stages given the known interindividual and intraindividual variance in hormone concentrations within a given pubertal stage.

### Statistical analysis

We first used descriptive statistics for assessing the completeness of data and, subsequently, potential selection bias. Specifically, we compared girls with regard to availability of stored sera (705, vs. 884 without) in relation to select sociodemographic and anthropometric characteristics. Next, we compared characteristics of girls with hormone measurements by inhibin B concentrations dichotomized as being below or above an *a priori* determined cutoff consistent with puberty (> 35 pg/mL) (Crofton et al. 2002; Sehested et al. 2000; Teilmann et al. 2007). Statistical significance was formally assessed using the chi-square (two-sided) test for categorical variables and weighted regression for continuous variables; *p*-values < 0.05 were considered significant and used for building the models. When comparing high and low inhibin B categories by anthropometric factors, we adjusted these measures for age for comparison to account for the strong age-related changes associated with puberty.

We used both descriptive and analytic techniques to inspect and assess the data for the 705 girls for whom stored residual sera were available for hormone measurement of which 689 (98%) had sufficient sample for inhibin B analysis. We excluded 34 in the “other” racial ethnic group owing to their small numbers, leaving 671 for the LH analysis and 655 for the inhibin B analysis. We used analysis of variance (ANOVA) to calculate least-square mean inhibin B concentrations (natural log transformed) by age and Pb categories for graphical presentation.

We used the survey procedures in SAS (version 9.1; SAS Institute Inc., Cary, NC) with sampling weights to account for the NHANES III complex sampling design to estimate effects of blood Pb on inhibin B concentrations. Survey logistic regression was used to estimate the association between whole-blood Pb and the prevalence of inhibin B (> 35 pg/mL) and LH (> 0.4 mIU/mL) above the *a priori* cutoffs. Both continuous (log-transformed) and categorical Pb variables (low Pb, < 1 μg/dL; moderate Pb, 1–4.9 μg/dL; and high Pb, ≥ 5 μg/dL) were modeled to account for the nonnormal distribution of blood Pb levels and evaluate potential nonlinearity. Although we are aware that Pb ≥ 5 μg/dL is not the conventional cutoff designating high Pb exposure, it was appropriate for our analyses given the distribution of blood Pb levels in our sample.

In complementary analyses [see Supplemental Material, Table 1 (doi:10.1289/ehp.1001943)], we used multinomial logistic regressions to estimate the association between continuous and categorical blood Pb (low, moderate, and high) and increasing categories of inhibin B [< LOD (60% of observations), medium (60–79th percentile), and high (≥ 80th percentile)] and LH [< LOD (48% of observations), medium (49–74th percentile), and high (≥ 75th percentile)]. Factors that altered the coefficient for Pb in multivariable models by > 10% were retained in the final multivariable models. Based on this criterion, body mass index (BMI; kilograms per square meter), race/ethnicity (non-Hispanic black and Mexican-American indicators, with non-Hispanic white as referent), census region (Midwest, South, and West indicators, with Northeast as referent), and PIR (indicators for categories < 1 and 1–2, with PIR > 2 as referent) were retained in all final multivariable models, and age (in months) was retained in the logistic models.

In the 40% of girls with levels of inhibin B above the LOD, we estimated the effect of urinary Cd on inhibin B and LH using multivariable survey regression, with Cd categorized into tertiles, using < LOD (automatically imputed as 0.01 ng/mL per NHANES III protocols; 33% of observations) as the reference level. We analyzed the effects of Cd and Pb classified as low Pb (< 5 μg/dL) and low Cd (first and second tertiles), low Pb and high Cd (third tertile), high Pb (≥ 5 μg/dL) and low Cd, and high Pb and high Cd. Likewise, we assessed the effects between moderate/high (≥ 1 μg/dL) and low Pb (< 1 μg/dL) and iron deficiency (yes/no). Although very few girls had iron deficiency in this sample, modeling was successful because the statistical weights allowed for estimation of category parameters.

Inhibin B did not demonstrate significant diurnal variation based on the available categories of blood draw timing. LH is known to have diurnal variation, specifically, “sleep-enhanced” variation near pubertal onset, but adjustment for timing of blood draw (morning, afternoon, evening) did not substantially alter associations among Pb, Cd, and LH.

## Results

Our sample comprised 705 girls 6–11 years of age with complete anthropometry (all girls) and Tanner staging data (for girls 8–11 years of age). After excluding 34 girls of “other” race/ethnicity due to small numbers, 671 and 655 girls had sufficient stored specimens available to measure LH and inhibin B, respectively. Compared with girls without available stored biospecimens, the study sample was slightly younger (by 4 months, *p* = 0.03), less likely to be classified as Tanner breast stage ≥ 3 (17% vs. 23%, *p* = 0.001) or non-Hispanic white (62% vs. 79%, p < 0.0001) or to live in a household with smokers (36% vs. 45%, *p* = 0.005). Girls with available samples also had lower Pb concentrations than did those with available sera [mean (95% CI), 0.7 μg/dL (0.6–0.9) vs. 0.9 μg/dL (0.8–1.02); *p* = 0.01]. Most anthropometric and hematologic characteristics did not significantly differ between the groups (data not shown).

The mean inhibin B concentration was 16.5 pg/mL [interquartile range (IQR), 4.95–20.2 pg/mL], with 14% of girls exceeding the inhibin B cutoff value of 35 pg/mL and 60% with values below the LOD. The mean LH concentration was 1.01 mIU/mL (IQR, 0.035–1.07 pg/mL), with 36% exceeding the LH cutoff value of 0.4 mIU/mL and 48% with values below the LOD.

We noted several differences between girls with inhibin B concentrations below and above 35 pg/mL. Girls with concentrations above the cutoff were more likely to be older (*p* < 0.0001) and taller (*p* < 0.0001) and had lower average BMI (*p* = 0.01) than did girls with concentrations below the cutoff (Table 1). Girls with concentrations > 35 pg/mL were significantly more likely to be Tanner stage ≥ 2 for both pubic hair and breast development and to live in the Northeast region (Table 2). The distribution of urinary Cd did not differ significantly by inhibin B, but girls with high inhibin B were more likely to have low blood Pb concentrations (13.5% vs. 28.4% < 1 μg/dL for girls with low inhibin B; *p* = 0.06) (Table 2). In addition, girls with concentrations > 35 μg/dL were somewhat less likely to have iron deficiency according to CDC criteria (0.1% vs. 1.8%, *p* = 0.09).

**Figure 1:**
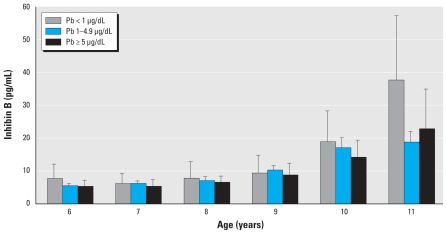
Inhibin B by whole-blood Pb level categories for girls, 6–11 years of age, from NHANES III, 1988–1994. Bars indicate least-square means, controlling for race/ethnicity; errors bars indicate 95% CIs.

The median whole-blood Pb concentration was 2.5 μg/dL (range, 0.07–29.4 μg/dL) with only 14% (*n* = 93) falling between 5 and 10 μg/dL and 5% (*n* = 32) exceeding 10 μg/dL, the CDC level of concern for children (Bellinger 2008). The median urinary Cd concentration was 0.12 ng/mL (range, 0.01–3.38 ng/mL). Non-Hispanic black girls had higher age-adjusted Pb levels (mean = 3.2 μg/dL) than did non-Hispanic white girls (mean = 1.8 μg/dL) and Mexican-American girls (mean = 2.4 μg/dL; *p* < 0.0001). Similarly, non-Hispanic black girls were also more likely to have high Cd levels (fourth quartile; 31.6%) than were non-Hispanic white girls (21.7%) or Mexican-American girls (18.3%; *p* = 0.002). Least-square mean values for inhibin B across the observed age range, by Pb category and controlling for race/ethnicity, indicate that older girls with low Pb measurements had higher inhibin B concentrations than did girls with moderate (1–4.99 μg/dL) or high Pb (≥ 5 μg/dL) measurements (Figure 1).

The likelihood of exceeding the inhibin B pubertal cutoffs was significantly decreased in association with a one-unit increase in log-transformed Pb concentration [adjusted odds ratio (OR) = 0.51; 95% CI, 0.34–0.78] (Table 3). Adjusted ORs for exceeding the pubertal threshold for inhibin B were 0.38 (95% CI, 0.12–1.15) and 0.26 (95% CI, 0.11–0.60) for girls with moderate and high Pb levels, respectively, compared with low levels. Adjustment for age, race/ethnicity, BMI, PIR, and census region tended to strengthen the magnitude of association away from the null.

Fifty-two percent of the 705 girls had detectable levels of LH, with 36% (*n* = 252) exceeding the pubertal cutoff value of 0.4 mIU/mL. Pb ≥ 5 μg/dL was inversely (though nonsignificantly) associated with LH above the cutoff [adjusted OR = 0.98 (95% CI, 0.48–1.99) and 0.83 (95% CI, 0.37–1.87) for moderate and high vs. low Pb, respectively]. Pb was not significantly associated with continuous LH concentrations before or after adjustment (data not shown).

Models examining the likelihood of exceeding increasing percentile categories for inhibin B and LH by blood Pb produced similar and confirmatory results [see Supplemental Material, Table 1 (doi:10.1289/ehp.1001943)], with significant inverse associations with blood Pb that were stronger for higher categories of inhibin B concentration and weaker nonsignificant associations with categories of LH concentrations. High Pb (≥ 5 μg/dL) compared with low Pb (< 1 μg/dL) was strongly inversely associated with having inhibin B ≥ 80th percentile compared with below the LOD (OR = 0.31; 95% CI, 0.12–0.83) but only moderately inversely associated with inhibin B between the 60th and 79th percentiles compared with below the LOD (OR = 0.70; 95% CI, 0.30–1.60). For LH, high Pb (≥ 5 μg/dL) compared with low Pb (< 1 μg/dL) was inversely associated with LH ≥ 75th percentile compared with below the LOD (OR = 0.48; 95% CI, 0.19–1.22), although not significantly so.

Urinary Cd (continuous or categorical) was not significantly associated with inhibin B or LH concentrations (data not shown). However, in the 40% of girls with inhibin B levels above the LOD, the inverse association with inhibin B concentrations was stronger for girls with high Cd and high Pb than for those with high Pb alone, with reference to girls with both low Pb and low Cd (Table 4). We also found a significant effect of iron deficiency and moderate/high Pb on inhibin B concentrations. Girls with moderate/high Pb (≥ 1 μg/dL) and iron deficiency had a significantly lower log inhibin B (β = −0.84; 95% CI, −0.39 to −1.29). In other words, the iron-deficient girls with even moderate levels of Pb exposure had even lower inhibin B concentrations than did iron-sufficient girls with moderate/high Pb concentrations (Table 4).

A sensitivity analysis restricted to girls with Pb levels < 10 μg/dL produced comparable results, although ORs were slightly attenuated. Given that girls with higher inhibin B tended to be slightly taller and thinner than girls with lower inhibin B, we explored an alternative hypothesis relating to reverse causality with regard to height. However, adjustment for height did not substantially alter the observed associations [see Supplemental Material, Technical Note 2 (doi:10.1289/ehp.1001943)].

## Discussion

As far as we are aware, this is the first study to identify a possible hormonal pathway by which Pb may delay pubertal onset and/or progression among girls participating in the NHANES III survey. Specifically, we observed that at older ages, girls with higher blood Pb concentrations had lower levels of inhibin B, and this was apparent even among girls whose Pb concentrations were below the 10-μg/dL value cited by the CDC as indicative of significant exposure in children. Additionally, having high Cd in addition to high Pb appeared to have an even greater impact on inhibin B levels than did high Pb alone. Two other noteworthy findings were that *a*) inhibin B levels were lowest for iron-deficient girls with moderate/high Pb levels, suggesting that Pb may be particularly toxic in iron-deficient girls, and *b*) there were racial/ethnic differences in Pb and Cd concentrations. Our findings add to a growing body of literature on the reproductive effects of environmental Pb and Cd exposure and extend the body of evidence to a large nationally representative sample of peripubertal girls.

In previous studies, blood Pb has been associated with later menarche in 1,706 girls 8–16 years of age who participated in NHANES III (Wu et al. 2003), and with later menarche among 138 Akwesasne girls 10–16.9 years of age (Denham et al. 2005). However, a smaller study of 87 9-year-old girls in New York City did not find an association between blood Pb and Tanner stage (1 vs. ≥ 2) (Wolff et al. 2008).

Animal studies are also consistent in finding that rats exposed to Pb have decreased LH and estradiol concentrations and a delay in sexual maturation (Dearth et al. 2002, 2004). Other studies have reported that Pb is associated with a dose-related delay in time to vaginal opening and decreased estradiol levels at puberty in rats exposed to environmentally relevant doses of Pb (Ronis et al. 1998a, 1998b).

The observed inverse association between Pb and inhibin B, a marker of follicular development through the peripubertal stages, adds further evidence that Pb may be associated with a delay of pubertal onset and the timing of its course. It has been hypothesized that reproductive and growth effects of Pb may involve multiple endocrine pathways (Ronis et al. 1996). A delicate balance of hypothalamic, pituitary, adrenal, and gonadal hormones and growth factors regulates the onset and tempo of sexual development, including multiple feedback mechanisms within the hypothalamic–pituitary–gonadal and hypothalamic–pituitary–adrenal axes, and then the onset is followed by a surge of gonadotropin secretion paralleling ovarian follicular development [reviewed by Buck Louis et al. (2008)]. Given that inhibin B is produced by the granulosa cells in the ovary (Hillier and Miro 1993), our results suggest that Pb may act directly at the level of the ovary, as well as acting through feedback mechanisms at the hypothalamic–pituitary level. In contrast, our results do not suggest that LH is affected by blood Pb at these ages, but an association between Pb and LH could be obscured due to interindividual and intraindividual variation in LH concentrations in this age group at or around the time of puberty (Manasco et al. 1997).

To our knowledge, there have been no previous studies of urinary Cd concentrations in relation to puberty timing in girls. In laboratory animals, Cd exposure has been associated with a decrease in estradiol (Zhang et al. 2008) and in LH and follicle-stimulating hormone (Paksy et al. 1989; Pillai et al. 2003). Cd also is known to accumulate in the granulosa cells of the ovary (Nampoothiri and Gupta 2006). It has been proposed that coexposure to Pb and Cd might exert a synergistic detrimental effect on reproductive performance in human pregnancy (Nampoothiri and Gupta 2006, 2008). Pb and Cd may act through similar physiologic and metabolic pathways, because both metals interact with and displace zinc and iron in different chemical reactions (Goyer 1997). Our finding that coexposure to higher levels of both Pb and Cd was associated with decreased inhibin B is consistent with the few animal studies of synergistic effects of Cd and Pb on hypothalamic–pituitary–adrenal function (Pillai et al. 2003; Tapisso et al. 2009) and their effects on bioavailability in humans (Dunicz-Sokolowska et al. 2006; Komjarova and Blust 2008; Osman et al. 1998). However, there is a paucity of research on the joint effects of metal mixtures on health outcomes, even though human exposure to metal mixtures occurs more often than exposure to isolated metals (Choudhury and Mudipalli 2008).

The observed effect of blood Pb ≥ 1 μg/dL, iron deficiency, and decreased inhibin B was striking. Although few girls in this U.S. sample were actually iron deficient, it is concerning that effects were apparent even at moderate levels of Pb exposure. It is known that children with high Pb exposure are also more likely to be iron deficient (Tripathi et al. 2001) and that gastrointestinal absorption of both Pb and Cd is increased in the presence of low iron stores (Goyer 1997; Osman et al. 1998; Tripathi et al. 2001). An effect of Pb and iron deficiency on inhibin B concentrations is plausible, given that both factors are known to independently affect health and cognition of children (Kordas et al. 2004). Iron-deficiency screening may be an important factor to consider as a part of blood Pb screening in at-risk populations and may be particularly important in less developed countries where the prevalence of iron deficiency is substantially higher than in the United States.

Environmental Pb exposure has decreased after implementation of the Lead Contamination Control Act of 1988 (amendment to the Safe Drinking Water Act) and legislation banning the sale of leaded gasoline for use in on-road vehicles, although children continue to have detectable levels of blood Pb, especially children from certain minority groups, low-income households, and internationally adopted children (CDC 2005). Consequently, subsequent national surveys probably will have relatively lower average Pb concentrations and may be less suited to address this etiologic question. The CDC cites Pb concentrations ≥ 10 μg/dL for toxic levels, yet health effects of neurodevelopmental deficits and growth impairments are observed for even modest levels of Pb, calling into question the existing threshold (Bellinger 2008; Ha et al. 2009). Furthermore, environmental Pb contamination remains a significant public health problem in developing countries. Pb concentrations in children from such locations as Mexico, India, Russia, and China are reported to be well above the CDC’s recommended threshold, with a range of 1–13% above the blood Pb threshold level of 10 μg/dL (CDC 2000; Tripathi et al. 2001).

NHANES III is a nationally representative sample of U.S. residents, making our results likely generalizable to the underlying U.S. population of girls who were 6–11 years of age between 1988 and 1994. However, our analytic sample comprised a subset of participants with stored sera available for additional laboratory analyses. Although we observed some differences between our study sample and those we excluded due to insufficient sera, including a slightly younger age and fewer non-Hispanic white girls, there is no reason to believe that the observed associations between Pb and inhibin B would differ substantially if we had included the entire NHANES III population. In fact, girls without stored sera had slightly higher mean blood Pb levels than did girls with stored sera, suggesting that the observed association between Pb and inhibin B would in fact have been more statistically significant because of increased statistical power if the entire population had been analyzed for a dose–response association. Furthermore, this study represents the largest study to date with hormonal measurements as markers of pubertal development in U.S. girls.

A limitation of this analysis includes the cross-sectional nature of the NHANES III data collection. Future studies should examine longitudinal hormone measurements to determine if Pb is associated with a delay in the increase of inhibin B and other hormonal markers consistent with the onset of puberty. Because of the limited amount of residual sera, we also were unable to measure additional hormones, such as estradiol and follicle-stimulating hormone, which might add further insight into the mechanism by which Pb may be associated with pubertal delay. Second, we cannot exclude the possibility that Pb and Cd exposures during prenatal, postnatal, or early childhood ages may explain or contribute to the observed decrease in inhibin B levels, rather than the levels observed at 6–11 years of age. Finally, we were compelled to estimate the magnitude of associations between Pb and the hormone cutoffs primarily using multinomial and logistic regression analyses because so many young girls at these ages had hormone levels below the LODs. We were thereby limited in our ability to detect subtle or linear effects (Schisterman et al. 2006).

## Conclusion

Our findings suggest a possible hormonal pathway by which blood Pb is associated with delayed onset and/or progression of puberty, that is, through a reduction in serum inhibin B or possibly through an interaction with Cd. Despite relatively low blood Pb levels in this sample of U.S. girls, we observed associations with pubertal hormonal markers, underscoring the importance of further efforts to reduce environmental Pb and Cd exposures.

## Figures and Tables

**Table 1:** Anthropometric characteristics of study sample by serum inhibin B pubertal cut point (35 pg/mL).

	Below		Above	
Characteristic	*n*	Weighted means (95% CI)	*n*	Weighted means (95% CI)
Age (years)	562	8.13 (8.00–8.25)	92	10.02 (9.70–10.34)
Height (cm)	563	134.1 (133.6–134.6)	92	137.1 (135.8–138.4)
Weight (kg)	561	32.9 (32.2–33.6)	92	32.1 (30.3–34.0)
BMI (kg/m^2^)	561	17.9 (17.6–18.1)	92	16.8 (16.1–17.6)

Age-adjusted least-square means and 95% CIs were calculated using weighted ANOVA.

**Table 2:** Distribution of sociodemographic and environmental characteristics by serum inhibin B pubertal cut point (35 pg/mL).

	Below		Above		
Variable	*n*	Percent (95% CI)	*n*	Percent (95% CI)	*p*-Value
Race/ethnicity					

Non-Hispanic white	125	61.7 (51.2–72.3)	15	57.4 (45.7–69.1)	0.60
Non-Hispanic black	214	23.4 (15.9–30.9)	42	28.9 (19.8–38.1)	
Mexican American	224	14.9 (9.6–20.1)	35	13.7 (6.8–20.6)	

PIR					

< 1	216	27.2 (19.3–35.2)	46	29.3 (18.7–39.9)	0.80
1–2	137	24.7 (18.5–30.8)	19	20.5 (12.9–27.9)	
> 2	163	48.2 (38.4–57.7)	24	50.3 (36.1–64.6)	

Census region					

Northeast	38	11.1 (4.1–18.1)	9	23.0 (17.4–28.3)	< 0.0001
Midwest	118	24.6 (15.6–33.6)	26	42.9 (30.2–55.3)	
South	225	37.6 (23.6–51.6)	36	21.4 (13.0–29.5)	
West	182	26.7 (11.4–42.1)	21	13.1 (8.0–18.3)	

Blood Pb (μg/dL)					

< 1	53	13.6 (7.6–19.5)	16	28.4 (10.4–46.6)	0.06
1–4.99	397	72.9 (66.1–79.6)	61	61.7 (43.3–79.9)	
≥ 5	111	13.6 (7.7–19.5)	14	9.9 (6.5–13.3)	

Urinary Cd (ng/mL)					

0.01–0.01	175	37.7 (29.8–45.6)	30	37.2 (21.0–53.4)	0.80
0.03–0.26	236	39.5 (31.7–47.4)	37	33.1 (13.5–52.8)	
0.27–3.38	120	22.8 (18.0–27.5)	20	29.7 (6.1–53.3)	

Pubic hair Tanner stage					

1	178	65.9 (57.1–74.8)	12	21.0 (11.2–30.9)	< 0.0001
2	66	19.9 (14.6–25.2)	24	31.3 (13.1–49.5)	
≥ 3	63	14.2 (8.5–19.9)	36	47.7 (20.4–75.0)	

Breast Tanner stage					

1	166	61.3 (51.7–71.0)	9	22.2 (12.4–32.0)	< 0.0001
2	89	27.5 (18.5–36.6)	28	34.1 (15.9–52.4)	
≥ 3	57	11.2 (6.4–15.9)	36	43.7 (16.4–70.9)	

**Table 3:** Association between blood Pb and odds of exceeding the inhibin B cutoff using weighted survey logistic regression.

Variable	*n*	OR (95% CI) Unadjusted	Multivariable adjusted^a^
Continuous Pb			

Log Pb	668	0.58 (0.36–0.95)	0.51 (0.34–0.78)

Categorical Pb			

Low, < 1 μg/dL	72	Reference	Reference
Moderate, 1–4.9 μg/dL	470	0.40 (0.14–1.15)	0.38 (0.12–1.15)
High, ≥ 5 μg/dL	126	0.35 (0.14–0.87)	0.26 (0.11–0.60)

a Adjusted for age (months), race/ethnicity (non-Hispanic white as referent), BMI (kg/m^2^), PIR (> 2 as referent), and census region (Northeast as referent).

**Table 4:** Effects of blood Pb and urinary Cd concentration, blood Pb, and iron deficiency on (natural log) inhibin B levels in 260 girls with inhibin B levels > LOD using weighted regression modeling.

	β-Value^a^ (95% CI)	*p*-Value
Pb/Cd category (*n* = 260)		

Low Pb (< 5 μg/dL) and low Cd (first and second tertiles)	Reference	
Low Pb (< 5 μg/dL) and high Cd (third tertile)	0.17 (–0.15 to 0.50)	0.29
High Pb (≥ 5 μg/dL) and low Cd (first and second tertiles)	−0.35 (−0.13 to −0.56)	0.002
High Pb (≥ 5 μg/dL) and high Cd (third tertile)	−0.52 (−0.07 to −0.97)	0.02

Pb/iron deficiency category (*n* = 260)		

Low Pb (< 1 μg/dL) and iron sufficient	Reference	
Low Pb (< 1 μg/dL) and iron deficient	−0.11 (0.32 to −0.53)	0.61
Moderate/high Pb (≥ 1 μg/dL) and iron sufficient	−0.39 (−0.06 to −0.71)	0.02
Moderate/high Pb (≥ 1 μg/dL) and iron deficient	−0.84 (−0.37 to −1.31)	0.0008

a Adjusted for race/ethnicity (non-Hispanic white as referent), BMI (kg/m^2^), PIR (> 2 as referent), and census region (Northeast as referent).
